# European Union training programme for tuberculosis laboratory experts: design, contribution and future direction

**DOI:** 10.1186/s12913-020-05240-3

**Published:** 2020-05-11

**Authors:** Philomena Raftery, Csaba Ködmön, Marieke J. van der Werf, Vladyslav Nikolayevskyy

**Affiliations:** 1 Department of Global Health and Development, Faculty of Public Health Policy, London School of Hygiene and Tropical Medicine, London, UK; 2 European Centre for Disease Prevention and Control, Stockholm, Sweden; 3 National Mycobacterium Reference Service South, Public Health England, London, UK

**Keywords:** Laboratory expert, Training Programme, Tuberculosis, Laboratory leadership

## Abstract

**Background:**

Tuberculosis (TB) control programmes rely heavily on laboratories to support both clinical care and public health. Qualified personnel with adequate technical and managerial skills comprise an integral component of any quality assured laboratory. Training a new generation of TB laboratory specialists was identified as a critical priority in the European Union /European Economic Area (EU/EEA). A tailored training programme for TB reference laboratory professionals was developed and implemented within the European Reference Laboratory Network for Tuberculosis to increase the pool of technical experts available to step into leadership roles in the TB laboratory community. Three cohorts of selected laboratory specialists participated in a series of trainings from 2009 to 2016.

**Methods:**

We conducted an evaluation of the training programme using a structured questionnaire administered via the EUSurvey website, with the aim of documenting the benefits and contribution as well as suggesting improvements and future direction of the programme. All graduated participants and all current ERLTB-Net members were invited to participate in the online survey and descriptive quantitative analysis was performed.

**Results:**

The evaluation found significant benefits for both the participants and the participants’ institutions, with improvements being reported in laboratory practices and management including implementation of new diagnostic techniques and career progression for participants. The training programme differed from other international and European initiatives in a number of important ways; the curriculum is unique in the scope and range of topics covered; the programme targets senior level professionals and future directors; cohorts were limited to 8–10 participants; and the programme involved a number of workshops (5–7) taking place over a two-year period. Relationships and collaborations established between individuals and institutions were valued as an important success of the initiative. Suggestions on how the impact of the programme could be enhanced included equipping participants to perform laboratory assessments in low-resource settings outside the EU, thus bolstering global TB control.

**Conclusion:**

Based on the findings presented the training programme has proved to be successful in developing leadership, expertise, partnerships and networks to support TB laboratories and has contributed significant benefits to strengthening European National Reference laboratories in the fight against TB.

## Background

Tuberculosis (TB) causes the highest number of deaths due to an infectious disease in the world, is the leading killer of people living with HIV and is associated with major challenges related to antimicrobial resistance [1]. In 2019, an estimated 10 million people developed TB and 1.5 million died from the disease worldwide, including 251,000 deaths among persons living with HIV [2]. Multidrug-resistant TB (MDR-TB) caused by bacilli resistant to at least two key antimicrobials (rifampicin and isoniazid) remains an emerging public health challenge globally [3, 4]. Accurate results from TB diagnostics and drug susceptibility tests enable TB programmes to identify TB cases and select appropriate treatment. Therefore, laboratories play a critical role in TB control both in supporting clinical care and public health activities: detecting active TB cases, monitoring treatment, contributing to outbreak investigations, contact tracing and surveillance activities [5–9].

Laboratories are complex systems comprising many components including infrastructure, equipment and technologies. Vital to their operation are the human resources and the systems that manage the processes and implement the standards to produce reliable, accurate and actionable results [10]. In addition, successful implementation of new and innovative diagnostic tests and technologies requires functional networks of laboratories with trained and motivated staff, robust quality management systems (QMS) and safe working environments. To achieve and maintain resilient laboratory systems, special consideration needs to be given to training programmes for laboratory management and leadership, strengthening laboratory networks through partnerships and pooling of resources, and developing strategies to integrate laboratories’ functions [10]. Qualified personnel with relevant technical and managerial skills comprise an integral component of a quality assured laboratory and are essential for obtaining and retaining laboratory accreditation [11]. A lack of personnel with advanced technical skills has been reported in many settings as a major obstacle to performing complex laboratory tests [5].

Existing TB laboratory training initiatives at the Global and European level have predominantly focussed on technical issues, QMS, planning and accreditation. The Stop TB Partnership’s Global Laboratory Initiative (GLI) [12, 13] developed a training package for TB diagnostic network planning which is predominantly administered through an online portal. This does not provide dedicated in-person practical training, which is vital for development of leadership and management skills, as well as for gaining expertise in conducting assessment visits. Another training offered by the European TB Laboratory Initiative (ELI) offers regional workshops that bring together laboratory specialists from countries with high TB and MDR-TB rates to strengthen their technical capacity in TB and MDR-TB diagnosis and implementing biosafety measures [14]. While these initiatives support the implementation of the World Health Organization (WHO) End-TB strategy [15] they do not specifically target the needs of senior level staff and laboratory managers serving as TB laboratory specialists in the EU/EEA. Thus, training of a new cadre of mycobacterial laboratory specialists was identified as an urgent requirement in the EU TB laboratory network [5, 16].

In order to strengthen the development of medium and senior level professionals, an innovative training programme was developed and implemented within the European Reference Laboratory Network for Tuberculosis (ERLTB-Net; ERLN-TB [before 2014]) from 2009 onwards [17]. The ERLTB-Net training programme aimed to increase the pool of technical experts with advanced knowledge of TB diagnostic methods, available to take leadership roles in national TB laboratories, and to support national TB laboratory systems and the EU/EEA TB diagnostic community. The curriculum for the training programme was developed by a group of TB experts experienced in providing advanced level training based on needs identified through External Quality Assessment (EQA) rounds [11] and decisions made at ERLTB-Net annual meetings. Between 2009 and 2016, the programme was provided to 22 laboratory specialists divided into three cohorts. All participants were subjected to a rigorous selection process. Trainees were first nominated by ERLTB-Net laboratories who selected them based on specific criteria including their work experience, level of seniority in their current position as well as potential benefits for the supporting laboratory. As potential candidates they then had to submit a portfolio including a CV and motivation letter highlighting their relevant experience and suitability as a trainee. Following shortlisting by the ERLTB-Net senior selection panel, participants were interviewed to confirm suitability before being enrolled as trainees.

The programme was delivered through a series of small (three to five per training cycle) and large workshops (two per training cycle) lasting 3 days each, with each training cycle spanning 2 years (see Additional file 1 for details of the topics covered). Small trainings were held at two ERLTB-Net member supranational TB reference laboratories fully accredited to ISO15189 standards located in the UK and Italy. These were predominantly laboratory-based and included both demonstration, interactive lectures and hands-on components covering basics in TB laboratory diagnosis, molecular diagnostic methods, latent TB diagnosis, TB epidemiology and contact tracing, laboratory assessment and TB laboratory design. Large trainings were combined with annual ERLTB-Net meetings and provided opportunities for developing networks, leadership, management and training skills through direct involvement of trainees in the meeting organization, teaching and discussions. Topics for large trainings included laboratory operations, TB policies and guidelines, laboratory financial management as well as emerging technologies in TB laboratory diagnosis. After completing the programme, graduates were involved in a variety of activities including workshops, taskforce visits, staff exchange visits and laboratory assessment visits.

All trainings were delivered by recognised experts in TB laboratory management, laboratory diagnostics, epidemiology and public health comprising of ERLTB-Net Laboratory Directors, senior staff members and external speakers from WHO, national public health bodies, and leading Universities. Following participant feedback from trainees of cohort one and two some modules and content were deemed less relevant or redundant for the target audience while other topics were incorporated into remaining modules so the course content was modified slightly. This demonstrated that participant feedback was incorporated at an early stage to improve the course and make it more relevant and impactful, otherwise training for all cohorts followed the same training curriculum developed as a part of ERLTB-net activities.

The goal of this study was to evaluate the training programme by exploring the benefits experienced by the participants and their supporting institutions throughout the period 2009–2016. We aimed to document the impact of the training on participants’ career development and the value of the training to the participants’ institutions. In addition, we sought to identify gaps and limitations of the training programme and make recommendations for improvement, development and its future direction.

## Methods

### Study design and setting

Two structured web-based questionnaires (see Additional files 2 and 3) were developed and distributed using an online platform. The first questionnaire was designed for participants who had completed the programme and consisted of 34 questions designed to evaluate their individual experience with the training. The questionnaire included questions on the application process, the quality of the training programme, participation in visits to other countries and perceived personal benefits. The second questionnaire was sent to heads of all ERLTB-Net member laboratories and consisted of 13 questions aimed at assessing the benefits of the programme from the perspective of the network members. An additional questionnaire (see Additional file 4) was circulated to all persons who participated in the training programme following initial analysis to augment information on the benefits of the training and the types of missions trainees had participated in.

Applying the methodology developed by Kilpatrick et al. to assess the perceived benefits of the training for the participants we compared initial expectations upon acceptance to the training programme to the level to which these expectations were met [18]. From a choice of 13 pre-defined answers (see Additional file 2), participants were asked to choose the four most relevant. Initial expectations were compared with the perceived principle gains or major self-reported achievements, to assess if the training programme allowed them to achieve additional skills versus original expectations. Objective benefits were assessed using a set of questions addressing individual’s career progression, process or system improvements, and new methodologies implemented in their home institutions.

### Participants

All individuals who had completed the programme between the years 2019–2016 (*N* = 22–1 deceased = 21) were invited to take part in the evaluation of the ERLTB-Net training programme along with all current ERLTB-Net members represented by heads of laboratories (*N* = 31).

### Data collection and analysis

The survey questions were uploaded to the EUSurvey (https://ec.europa.eu/eusurvey/) website, a web-based tool, in order to allow easy distribution, access and responses submission for all respondents with the option to remain anonymous. Upon completion of data collection, which lasted two months, the responses were exported to Microsoft Excel (Microsoft Corporation, Redmond, Washington) spreadsheets for compilation of the data sets followed by descriptive quantitative analysis.

## Results

### Characteristics of the respondents

Response rates to the questionnaires were 85.7% (18/21 training participants) and 58.0% (18/31 heads of TB laboratories). Of the 18 respondents to the participant questionnaire, five had been trained in cohort 1, seven in cohort 2 and six in cohort 3. The highest level of education at the time of application to the training was Bachelor of Science degree (BSc) 17%, Master of Science (MSc) 33%, and Doctor of Philosophy (PhD) 50%. Seventy-two percent of the respondents were > 35 years old, 17% within the age range 31–35 years and 11% in the range 25–30 years old. The level of seniority at the time of application, judged as an individuals’ position within their organisation, ranged between intermediate (39%) and senior level (61%). Of the 18 participants, 15 (84%) were employed at a National Reference Laboratory (NRL) at the time of application while 2 (11%) were working at a Supra- National Reference Laboratory (SNRL) and one respondent worked at a clinical laboratory. The majority of applicants had some knowledge about the training programme prior to application, with 88% reporting that the institution in which they worked was a member of the network, and/or they had colleagues who had already participated in the training programme. All applicants reported that they were adequately supported by their home institution throughout the application process.

### Subjective benefits

**Fig. 1:**
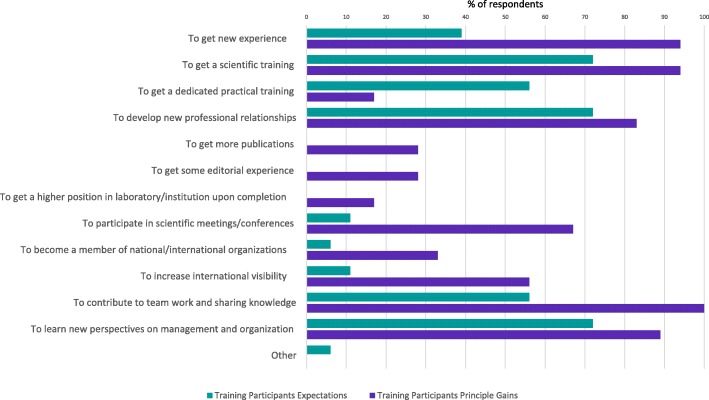
Comparison of initial expectations with principle gains of training participants expressed in % of respondents

The key expectations of the training programme included receiving scientific training, development of professional relationships and building managerial expertise (*n* = 13 respondents). The self-reported achievements of the training participants were generally in agreement with their expectations, with scientific training, development of professional relationships, managerial expertise, networking skills and participation in conferences being the achievements reported by most participants (Fig. 1).

In all but one category (dedicated practical training), the principle gains reported surpassed the participants’ initial expectations. In addition to what was expected from the training programme, participants learned to work in teams (18 respondents), to share expertise (17 respondents) and received scientific training in TB diagnostic and research methodologies (17 respondents). Although categories “get more publications”; “get some editorial experience”; and “get a higher position” had not been selected as initial expectations by any participant, these were self-reported as achievements by five, five, and three participants, respectively.

**Fig. 2:**
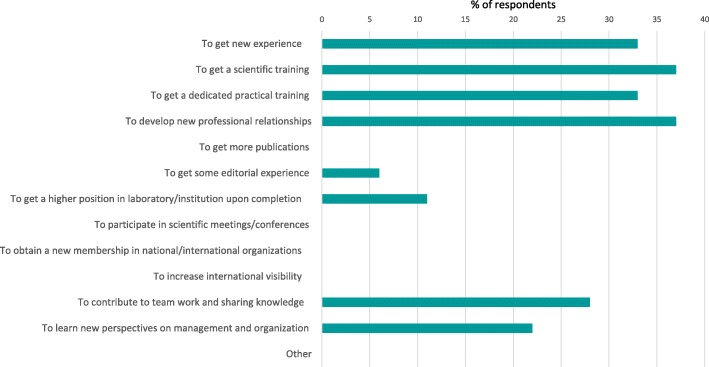
Expectations of European Reference Laboratory for TB Network members expressed in % of respondents

ERLTB-Net members (*N* = 18) that had nominated staff to participate in the programme gave similar responses to those of the training participants regarding initial expectations (Fig. 2). The majority of the network members cited the following six reasons for supporting the application of their staff member: to get new experience, to get a scientific training, to get a dedicated practical training, to develop new professional relationships, to contribute to team work and sharing knowledge with new colleagues and peers and to learn new perspectives on management and organization of TB reference laboratories and national laboratory networks.

### Benefits for training participants

All respondents reported that the ERLTB-Net training empowered them to improve the diagnostic services in their institution, expanded their expertise in advanced TB diagnostics, and facilitated their career progression.

Of the 18 respondents, nine (50%) had progressed to a more senior position since completion of the training programme; seven had been promoted to a more senior position in the same institution, one had moved to a more senior position in a different public health institution in the same country and one had obtained a more senior position in an international public health organisation. Nearly three quarters (72%) of respondents reported that they had established links with other institutions and initiated information sharing, expertise sharing and/or new projects through collaborations made during the training programme.

**Fig. 3:**
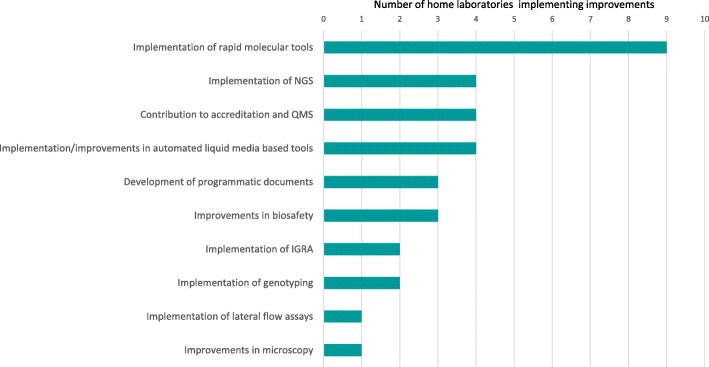
Improvements implemented by training participants (*N* = 18) in home institutions following completion of the training programme

Following graduation, new methods, policies and practices had been introduced in all laboratories with many laboratories implementing more than one change (Fig. 3). Changes ranged from improvements in methodologies, implementation of additional techniques such as liquid culture, liquid culture for drug-susceptibility testing (DST), molecular identification, molecular DST, interferon-gamma release assay (IGRA), epidemiological genotyping, next generation sequencing (NGS) and improvements to the QMS, biosafety, training and diagnostic algorithms, guidelines and policies.

Laboratory directors were asked to grade (1–10) the perceived gains for their institution of having a staff member participating in the training programme. Areas which respondents graded > 5 included: team work and sharing knowledge with new colleagues and peers, implementation of new practices, increased international visibility, participation in joint projects with other institution, new perspectives on TB laboratory organization and activity, implementing new methods/protocols and guidelines/policies and becoming members of national/international organizations. Building partnerships and collaborations between participating laboratories was considered a key benefit of the training and one which warranted further investment.

“In my opinion there must be more focus on the twinning programmes between countries. Also, relationships already established between labs should be further supported.” (ERLTB-Net member).

### Participation in missions

Ten of the 18 graduated training participants reported having taken part in one or more country and/or laboratory assessment missions. All of these were conducted at NRL’s with the duration of missions varying from 1 to 3 days. The types of missions ranged from joint ECDC/WHO-Europe missions, ERLTB-Net commissioned task force visits and exchanges, missions to regional laboratories and international WHO commissioned missions to laboratories in Africa. Participating in missions was seen by support experts as a key way for trainees to apply their knowledge in a tangible way. This was also highlighted as an important learning opportunity for those coming from more advanced laboratories on the diverse contextual challenges and resource limitations experienced in different countries across the region.

“If funding would be available, it would be great to make. .. a mission available for each support expert during training... . you really grow as a person after such mission. At the same time you’ll be very humble by seeing the difference in possibilities of various labs, due to aspects like economy, biosafety, burden of MDR-TB patients and so on.” (newly trained Support Expert).

Of the 18 heads of TB laboratories participating in the network survey, five had hosted a mission by a training participant. Outputs received following the mission included post-visit debriefing, written reports, lists of recommendations and support provided on implementation of recommendations. A high proportion (80%) of laboratories who had hosted a mission reported making changes or taking action based on the findings of the mission. Members reinforced the point that newly trained support experts should be afforded the opportunity to participate in missions and that the training should be continued for additional cohorts.

### Satisfaction levels and suggested improvements

The majority of respondents (91.0–100% for different training events) felt that the training content was relevant and within the scope of the training program with overall satisfaction levels reaching 75.0–100% of participants. Suggestions on how the training programme could be improved or optimised included making the programme longer and expanding it to cover additional topics including new technologies, addressing emerging problems like migration and its implications for TB diagnostic services, development of networking capabilities and expanding research and education opportunities (see Additional file 5).

“Should look at how research collaborations can be built between countries and encourage SNRL’s to support further education of colleagues at NRL’s” (newly trained Support Expert).

Sixty-eight percent of the ERLTB-Net members were satisfied with the programme and recommended changes included: utilising existing collaborations and twin arrangements between the laboratories, concentrating on developing practical skills, support for research collaborations and implementation of online training (see Additional file 6). Some support experts suggested advocating for the training programme to be recognised by WHO so that support experts within the network could be called on as additional expertise to bolster Global TB efforts.

“Training should allow support experts to perform assessments with WHO outside the European context in the developing world where the need is greatest” (newly trained Support Expert).

An important recommendation which was highlighted by a number of training participants was that newly trained support experts should be involved more in leadership and management of the network activities and that opportunities to participate in the ERLTB-Net annual meetings should be expanded.

“I think younger experts should be encouraged to participate more in the activities of the management team decision and policy discussions and to broaden the core group beyond the more senior experts” (newly trained Support Expert).

## Discussion

Resilient laboratory systems are critical to support TB programmes, infectious disease diagnostics and public health systems. Global Health crises such as the current Coronavirus disease 2019 (COVID-19) Pandemic and the 2014–16 West Africa Ebola outbreak highlight the importance of functional laboratory systems and diagnostic capabilities [19–22]. Unfortunately, laboratory systems and infrastructure have long been neglected and under-resourced in many settings. Investment in training and upskilling of human resources should be considered a critical component of laboratory system strengthening. Recognising this need in the EU Region, the ERLTB-Net set out a clear objective to invest in developing a cadre of future leaders within the TB diagnostic community.

A tailored training programme for TB reference laboratory experts was developed and implemented within ERLTB-Net to address the specific needs of the EU/EEA Member States [17]. The ultimate goal of the training programme was to increase the pool of technical experts available to step into leadership roles in national TB laboratories and in the EU/EEA international TB diagnostic community. The course specifically targeted future leaders in TB diagnostics in the European region, therefore recruited those likely to take up management and leadership positions in TB laboratories within the EU, to achieve optimal impact. A total of 21 persons were trained within this programme in 2009–2016 and many of them subsequently took up leadership roles in TB laboratories. A high proportion of training participants reported having been promoted to more senior positions nationally and internationally following their graduation, indicating that the training program was beneficial for their career progression and fulfilling one of the fundamental objectives of the training programme. Satisfaction with the training programme was generally high and self-reported achievements exceeded initial expectations in the vast majority of categories, indicating significant perceived benefits for the trainees. Practical training was the only area where expectations were high but gains were relatively low (Fig. 1) which could be explained by the fact that the advanced training covered various aspects of which hands on training was just one component. Inclusion of multiple components spanning theory and practice, combined with leadership and management aspects, allowed trainees to gain a broader expertise which is demonstrated by significant gains in other areas.

The course was offered to TB NRL’s in the entire European region where the vast disparity in public health development, available resources and TB diagnostic capabilities is evident between laboratories in these countries. One of the significant objectives and benefits of the ERLTB-Network is to act as a platform to coordinate capacity building of developing NRL’s by other high-resource reference laboratories. Our findings demonstrate the critical opportunities available for more advanced NRL’s and SNRL’s in the European region to share their experiences and support development of those operating in low-resource contexts through missions and collaborations. Furthermore, an important objective of this training programme was to harmonize diagnostic methodologies across the European region. Various new methods, policies and practices have been implemented in participants’ home institutions following graduation, achieving a key goal of the programme. Recently Mathys et al. [23], demonstrated the value of networking activities in sharing expertise and developing methodologies that could be used to improve quality and laboratory performance within the ERLTB-Net and beyond. Reported benefits for the ERLTB-Net further supported these findings indicating that the programme had a positive influence on the TB Laboratory network at ground level.

It is widely accepted that to help ensure that laboratories can effectively play their critical role in the detection, prevention and control of diseases, laboratory directors and senior laboratory managers require specialised training in leadership and management. To achieve this, in 2020, WHO expect to launch a new training programme the Global Laboratory Leadership Programme (GLLP) which is anticipated to greatly contribute to the laboratory profession globally. However, the content and teaching modalities are still under development and it is unclear if the programme will be administered in person or through distance learning [24]. While there are currently a number of other training initiatives available to support capacity building of laboratory staff, the ERLTB-Net training programme which has been running for more than a decade, differs from those available in a number of important ways [13, 14]. Firstly, the training curriculum is unique in the scope and range of topics covered within the programme which includes education on TB policy and guidelines; management, leadership and organisation of TB laboratories; technical training on new methodologies, as well as QMS and biosafety issues. Secondly, the programme is pitched at a very high level, targeting future directors and senior level professionals in TB diagnostics in the European region, which has been identified as a critical need in the EU/EEA area. The requirement for training of senior TB experts was further supported by the results of a recent survey (ERLTB-Net, unpublished data) demonstrating that 32.0% and 61.3% of senior technicians and heads of national TB reference laboratories in EU/EEA countries are approaching retirement age (50 years +) or are above retirement age. In countries that joined the EU in 2004 and later these proportions are even higher (42.1% and 63.6%, respectively). The recruitment process for this training involved careful selection of participants based on their long-term commitments, qualifications and nominations from host institutions, prioritising laboratory experts already working in NRLs and SNRLs. Thirdly, training cohorts are purposely limited in number with only eight to ten participants per cohort facilitating targeted training, fostering relationships and encouraging collaborations to be built over the period of the training. Finally, the programme took place at a number of venues within the EU (UK and Italy), with trainees participating in a number of small and large workshops spread over the two-year period. This longitudinal nature of training (as opposed to one off training offered by many initiatives) further facilitated network and relationship building, resulting in collaborations and partnerships developing, with many subsequent positive outcomes.

A high proportion (80%) of laboratories who had hosted a visit by a support expert reported making changes or taking action based on the findings of the visit, demonstrating the usefulness of the exercise. The findings clearly showed that support experts were enthusiastic to contribute to and participate in missions. Participating in missions was seen by support experts as a key way for trainees to galvanize and apply their learning, and to contribute to capacity building and harmonization of systems and practices across the European region. An important suggestion on how the impact of the programme could be enhanced was that graduated experts should also be trained in performing assessments beyond the European context in low-resource settings where the need is greatest, enabling the training programme to support global TB control.

All of the respondents reported that they would recommend colleagues to apply for the training programme and made a number of important suggestions on how the programme could be improved which are being explored by the training coordinators for future cohorts. A notable suggestion was that the programme should be used to promote research collaborations between countries and encourage SNRLs to support further education of colleagues at NRLs which would ultimately benefit the ERLTB-Net. An additional suggestion included ensuring that trained experts are continually involved in the work of the Network and in future training and meetings. Evidence that this is already being implemented was visible at recent annual meetings where a number of graduated experts contributed in both organisational and teaching components. Suggestions for future provision of online training courses, additional practical training and further supporting relationships and collaborations are also being explored.

### Limitations

A number of limitations exist relating to the methodology of this study, including that the responses and therefore results and conclusions are based on self-reported participants’ opinion. Evaluations in the form of pre- and post- tests were not performed and results of post-training evaluations were not included in the analysis presented here. Additionally, the information about expectations was collected retrospectively and not before the start of the training programme. We also highlight the low response rate to the ERLTB-Network questionnaire (58%). In addition, we did not gather information on other training courses attended by the trainees which could have contributed to or augmented the benefits and career progression of trainees which we report here.

## Conclusions

Based on the finding presented and discussed above we believe that the concept of the ERLTB-Net training programme has proved to be successful in developing expertise, partnerships and networks to support TB laboratories in the EU/EEA and has contributed significant benefits to European NRLs in the fight against TB. This manuscript describes an important initiative with demonstrated successes of career progression, improvements in technical skills and competencies, development of collaborations and value added to the broader TB community. We propose that the training programme described here would be extremely beneficial if implemented within other regions outside the EU, for example in Africa and Asia, where TB prevalence is high and laboratory systems are weak and traditionally neglected. Now more than ever, public health programmes would benefit from dedicated experts trained to lead and manage TB and infectious disease laboratories and this manuscript describes a potential framework for implementation.

## Supplementary information

**Additional file 1.** Details of the topics covered during each training course.

**Additional file 2.** Questionnaire for ERLTB-Net Support Experts.

**Additional file 3.** Questionnaire on ERTB-Net Support Experts training programme benefits for the network and TB community as a whole.

**Additional file 4.** Additional questionnaire on Missions and benefits for the laboratories for ERLTB-Network members and trained Support Experts.

**Additional file 5.** Additional topics suggested by participants for inclusion in the training programme.

**Additional file 6.** Proposals from ERLTB-Net members to enhance the training programme.

## Data Availability

The datasets analysed as part of the current study are available from the corresponding author on reasonable request.

## Abbreviations

DST: Drug-susceptibility testing
ELI: European TB Laboratory Initiative
EQA: External Quality Assessment
ERLTB-Net: European Reference Laboratory Network for Tuberculosis
EU/EEA: European Union/European Economic Area countries
GLI: Global Laboratory Initiative
IGRA: Interferon-gamma release assay
MSc: Master of Science
NRL: National Reference Laboratory
PhD: Doctor of Philosophy
QMS: Quality Management Systems
SNRL: Supra- National Reference Laboratory
TB: Tuberculosis
WHO: World Health Organization
