# Supplementary material for: European Union training programme for tuberculosis laboratory experts: design, contribution and future direction
Source: BMC Health Serv Res. 2020 May 11;20:413. doi: 10.1186/s12913-020-05240-3 (PMC7212721; doi:10.1186/s12913-020-05240-3)
Supplement: Supplementary file 1 — Additional file 1. Details of the topics covered during each training course. [file 12913_2020_5240_MOESM1_ESM.docx]

# Additional Information

# Additional File 1:

**Topics covered during the two-year European Reference Laboratory for Tuberculosis Network Training Programme**

| **Topics covered** |
| --- |
| **Cohorts 1 and 2 (12 Respondents)** |
| **Small training workshop 1 - Basics in TB laboratory diagnosis** |
| **Small training workshop 2 - Molecular methods in TB laboratory diagnosis** |
| **Small training workshop 3 - Latent TB diagnosis and contact tracing** |
| **Small training workshop 4 - Laboratory assessment visits** |
| **Small training workshop 5 - TB laboratory design and financial management** |
| **Cohort 3 (6 Respondents)** |
| **Small training workshop 1 - Basics in TB laboratory diagnosis** |
| **Small training workshop 2 - Molecular methods and emerging technologies in TB laboratory diagnosis** |
| **Small training workshop 3 - Laboratory assessment visits** |
| **ALL cohorts (18 Respondents)** |
| **Large training workshop 1 (Annual meeting Year 1)** |
| **Large training workshop 2 (Annual meeting Year 2)** |
