# Supplementary material for: European Union training programme for tuberculosis laboratory experts: design, contribution and future direction
Source: BMC Health Serv Res. 2020 May 11;20:413. doi: 10.1186/s12913-020-05240-3 (PMC7212721; doi:10.1186/s12913-020-05240-3)
Supplement: Supplementary file 2 — Additional file 2. Questionnaire for ERLTB-Net Support Experts. [file 12913_2020_5240_MOESM2_ESM.docx]

# Additional File 2:

# Questionnaire for ERLTB-New Support Experts

1. Your name and surname (optional)
2. Name of laboratory and country you are currently working in
3. What is your current position?
4. How do you define level of seniority of the position you are currently holding?
   1. Senior
   2. Intermediate
   3. Entry level
5. Your age at the time of application?
6. <25
7. 25-30
8. 31-35
9. >35
10. Which cohort were you part of (Cohort, Year)
11. 1, 2009-10
12. 2, 2011-13
13. 3, 2014-15
14. Your highest level of education at the time of application to participate in the training programme
15. Degree
16. Masters
17. PhD
18. Other, please specify _______________________________

**APPLICATION PROCESS**

1. How did you know about Support Expert Training programme?
   1. From the ERLTB-Net extranet website;
   2. From your supervisor / senior staff at your home institution;
   3. From your colleagues;
   4. Heard about it at the ESM congress
   5. Other (please specify)_______________________________
2. What information did you have on the training prior to application
   1. My laboratory was a member of ERLTB-Net
   2. My colleague was participating in Support Expert training
   3. Had never heard of ERLTB-Net or of Support Expert training
3. Did you get enough support from your home institution in the application process?
   1. Yes
   2. No
4. What was the level of the laboratory you worked in at the time of application
5. National Reference Laboratory
6. Regional Reference Laboratory
7. Clinical laboratory
8. University/Research laboratory
9. Other (please specify) ­­­­­­­­­­­­­­­_______________________________
10. Did you have work colleagues who were trained as support experts in previous cohorts
    1. Yes
    2. No

**QUALITY OF TRAINING**

1. What were your initial expectations upon acceptance to the training course?

1. To get new experience;
   1. Yes
   2. No
   3. Don’t know
2. To get a scientific training (courses, seminars, congresses, greater access to training material etc.);
   1. Yes
   2. No
   3. Don’t know
3. To get a dedicated practical training (new or improved skills in performing and/or interpreting advanced techniques, new accreditations etc.);
   1. Yes
   2. No
   3. Don’t know
4. To develop new professional relationships (joint projects with other institutions, relationship with other scientific co-operations and networks etc.);
   1. Yes
   2. No
   3. Don’t know
5. To get more publications (papers, abstracts, book chapters etc.);
   1. Yes
   2. No
   3. Don’t know
6. To get some editorial experience (drafting, submitting and completing revisions of manuscripts etc.);
   1. Yes
   2. No
   3. Don’t know
7. To allow the staff member to get a higher position in laboratory/institution upon completion;
   1. Yes
   2. No
   3. Don’t know
8. To participate in scientific meetings/conferences (oral abstract presentations, invited lectures etc.);
   1. Yes
   2. No
   3. Don’t know
9. To obtain a new membership in national/international organizations;
   1. Yes
   2. No
   3. Don’t know
10. To increase international visibility;
    1. Yes
    2. No
    3. Don’t know
11. To contribute to team work and sharing knowledge with new colleagues and peers;
    1. Yes
    2. No
    3. Don’t know
12. To learn new perspectives on management and organization of TB reference laboratory and national laboratory network (staff management, reporting, quality control etc.);
    1. Yes
    2. No
    3. Don’t know
13. Other (please, specify)________________________________
14. Were these expectations met?
15. To get new experience;
    1. Fully
    2. Partially
    3. Not at all
16. To get a scientific training (courses, seminars, congresses, greater access to training material etc.);
    1. Fully
    2. Partially
    3. Not at all
17. To get a dedicated practical training (new or improved skills in performing and/or interpreting advanced techniques, new accreditations etc.);
    1. Fully
    2. Partially
    3. Not at all
18. To develop new professional relationships (joint projects with other institutions, relationship with other scientific co-operations and networks etc.);
    1. Fully
    2. Partially
    3. Not at all
19. To get more publications (papers, abstracts, book chapters etc.);
    1. Fully
    2. Partially
    3. Not at all
20. To get some editorial experience (drafting, submitting and completing revisions of manuscripts etc.);
    1. Fully
    2. Partially
    3. Not at all
21. To allow the staff member to get a higher position in laboratory/institution upon completion;
    1. Fully
    2. Partially
    3. Not at all
22. To participate in scientific meetings/conferences (oral abstract presentations, invited lectures etc.);
    1. Fully
    2. Partially
    3. Not at all
23. To obtain a new membership in national/international organizations;
    1. Fully
    2. Partially
    3. Not at all
24. To increase international visibility;
    1. Fully
    2. Partially
    3. Not at all
25. To contribute to team work and sharing knowledge with new colleagues and peers;
    1. Fully
    2. Partially
    3. Not at all
26. To learn new perspectives on management and organization of TB reference laboratory and national laboratory network (staff management, reporting, quality control etc.);
    1. Fully
    2. Partially
    3. Not at all
27. Other (please, specify)________________________________
    1. Fully
    2. Partially
    3. Not at all
28. Please provide details of the training courses attended and your opinion on the quality of teaching and content by filling in the following table

| **Course attended** | **Did you find training relevant (Yes/No)** | **Were you satisfied with the training (Fully/partially/not at all)** | **How much of the new knowledge did you apply in your place of work following the training** |
| --- | --- | --- | --- |
| Cohorts 1 and 2 ONLY |  |  |  |
| Small training workshop 1 |  |  |  |
| Small training workshop 2 |  |  |  |
| Small training workshop 3 |  |  |  |
| Small training workshop 4 |  |  |  |
| Small training workshop 5 |  |  |  |
| Cohort 3 ONLY |  |  |  |
| Small training workshop 1 |  |  |  |
| Small training workshop 2 |  |  |  |
| Small training workshop 3 |  |  |  |
| ALL cohorts |  |  |  |
| Large training workshop 1 (combined with annual meeting) |  |  |  |
| Large training workshop 2 (combined with annual meeting) |  |  |  |

1. How could training courses be improved or optimised (up to three answers may be selected)
2. Make it longer
3. Make it shorter
4. Expand content to cover more topics
5. Decrease content to cover less topics
6. Make the groups larger
7. Make the groups smaller
8. Other (specify) ________________________________
9. What additional topics do you think should have been included in the course content

Pease specify: ________________________________

**PARTICIPATION IN MISSIONS**

1. How many missions have you taken part in
   1. None
   2. One
   3. Two
   4. Three
   5. More than three
2. How many assessors formed part of your team
   1. One
   2. Two
   3. Three
   4. More than three
3. In what country(s) did you perform the mission

_______________________________

1. What was the level of the laboratory visited by you and your team
2. National Reference Laboratory
3. Regional Reference Laboratory
4. Clinical laboratory
5. Research laboratory
6. Other (specify) _______________________________
7. What was the duration of the mission
   1. One day
   2. Two days
   3. Three days
   4. More than three days
8. For the support experts who participated in a mission, what were the outputs of the mission
   1. Written Report
   2. Verbal Report
   3. List of Recommendations
   4. Support provided on implementation of recommendations
   5. None
   6. Other (specify) ­­­­­­­­­_____________________________________

**PERSONAL BENEFITS**

1. In general, you feel your skills and knowledge past graduation:
2. Exceeded your aims and expectations,
3. Were in agreement with your aims and expectations,
4. Appeared to be below your aims and expectations
5. How would you describe your principal gains after completion of the Support Expert training.
6. New experience;
   1. Yes
   2. No
   3. Don’t know
7. Scientific training (courses, seminars, congresses, greater access to training material etc.);
   1. Yes
   2. No
   3. Don’t know
8. Dedicated practical training (new or improved skills in performing and/or interpreting advanced techniques, new accreditations etc.);
   1. Yes
   2. No
   3. Don’t know
9. Development of new professional relationships (joint projects with other institutions, relationship with other scientific co-operations and networks etc.);
   1. Yes
   2. No
   3. Don’t know
10. More publications (papers, abstracts, book chapters etc.);
    1. Yes
    2. No
    3. Don’t know
11. Some editorial experience (drafting, submitting and completing revisions of manuscripts etc.);
    1. Yes
    2. No
    3. Don’t know
12. Getting a higher position in laboratory/institution upon completion;
    1. Yes
    2. No
    3. Don’t know
13. Participation in scientific meetings/conferences (oral abstract presentations, invited lectures etc.);
    1. Yes
    2. No
    3. Don’t know
14. Obtaining a new membership in national/international organizations;
    1. Yes
    2. No
    3. Don’t know
15. Increase in international visibility;
    1. Yes
    2. No
    3. Don’t know
16. Contribution to team work and sharing knowledge with new colleagues and peers;
    1. Yes
    2. No
    3. Don’t know
17. Knowledge of new perspectives on management and organization of TB reference laboratory and national laboratory network (staff management, reporting, quality control etc.);
    1. Yes
    2. No
    3. Don’t know
18. Other (please, specify)________________________________
19. What did you manage to implement in your home institution after finishing your training. More than one answer can be selected,
20. New methods/protocols;
21. New practices;
22. New guidelines;
23. New policies;
24. Participated in joint projects with other institution;
25. other (please specify)_____________________________________________
26. Did your example motivate other your colleague(s) to apply for the Support Expert training
27. Yes
28. No
29. I don’t know
30. Did you establish links with other institutions and initiated information sharing, expertise sharing, new projects
31. Yes
32. No
33. In your opinion, did becoming a support expert influence your future career plans
34. Yes
35. No
36. In which way(s) did becoming a support expert influence your future career plans. Up to three answers can be selected
37. To improve the diagnostic services in your institution;
38. To improve the research activity in your institution;
39. To apply for further study (MSc/PhD;
40. To improve the expertise in advanced TB diagnostics;
41. To assist your laboratory to apply for/achieve accreditation
42. To participate in ERLTB-Net activities;
43. To change your career path;
44. Other (please specify)____________________________________________
45. At the moment do you work at:
46. The same institution - more senior position,
47. The same institution - the same position,
48. other healthcare organization in your home country,
49. international healthcare organization,
50. other (please specify)_________________________________________
51. Did the training help with your career progression or increase your chances to get a better position in your institution
52. Yes
53. No
54. Don’t know

If yes, in what way?

1. Promotion to a senior position
2. Getting a permanent position
3. Other (please specify)
4. Would you recommend other colleague(s) to apply for the Support Expert training
5. Yes
6. No
7. I don’t know
8. In your opinion, how could the existing programme be improved _____________________________________________________________
9. Do you have any further comments and/or suggestions about the training course

_______________________________
