# Supplementary material for: European Union training programme for tuberculosis laboratory experts: design, contribution and future direction
Source: BMC Health Serv Res. 2020 May 11;20:413. doi: 10.1186/s12913-020-05240-3 (PMC7212721; doi:10.1186/s12913-020-05240-3)
Supplement: Supplementary file 3 — Additional file 3. Questionnaire on ERTB-Net Support Experts training programme benefits for the network and TB community as a whole. [file 12913_2020_5240_MOESM3_ESM.docx]

# Additional File 3:

# Questionnaire on ERTB-Net Support Experts training programme benefits for the network and TB community as a whole

1. Your name and surname (optional)
2. Name of laboratory and country you are currently working in
3. What is your current position?
4. How do you define level of seniority of the position you are currently holding?
   1. Senior
   2. Intermediate
   3. Entry level
5. Has a staff member from your institution been nominated to train as a support expert?
   1. Yes
   2. No

IF YOU HAVE ANSWERED YES PLEASE GO TO QUESTION 6

IF YOU HAVE ANSWERED NO PLEASE GO TO QUESTION 9

1. What was your reason for supporting the staff member?

1. To get new experience;
   1. Yes
   2. No
   3. Don’t know
2. To get a scientific training (courses, seminars, congresses, greater access to training material etc.);
   1. Yes
   2. No
   3. Don’t know
3. To get a dedicated practical training (new or improved skills in performing and/or interpreting advanced techniques, new accreditations etc.);
   1. Yes
   2. No
   3. Don’t know
4. To develop new professional relationships (joint projects with other institutions, relationship with other scientific co-operations and networks etc.);
   1. Yes
   2. No
   3. Don’t know
5. To get more publications (papers, abstracts, book chapters etc.);
   1. Yes
   2. No
   3. Don’t know
6. To get some editorial experience (drafting, submitting and completing revisions of manuscripts etc.);
   1. Yes
   2. No
   3. Don’t know
7. To allow the staff member to get a higher position in laboratory/institution upon completion;
   1. Yes
   2. No
   3. Don’t know
8. To participate in scientific meetings/conferences (oral abstract presentations, invited lectures etc.);
   1. Yes
   2. No
   3. Don’t know
9. To obtain a new membership in national/international organizations;
   1. Yes
   2. No
   3. Don’t know
10. To increase international visibility;
    1. Yes
    2. No
    3. Don’t know
11. To contribute to team work and sharing knowledge with new colleagues and peers;
    1. Yes
    2. No
    3. Don’t know
12. To learn new perspectives on management and organization of TB reference laboratory and national laboratory network (staff management, reporting, quality control etc.);
    1. Yes
    2. No
    3. Don’t know
13. Other (please, specify)________________________________
14. Was supporting a staff member to be trained as a support expert included as part of your laboratory staff development plan?
    1. Yes
    2. No
15. What did the institution gain from the Support Expert training? Please rate each question 1 to 10 (0 – no gains, 10 – significant gains)

- 1. Implemented new methods/protocols;
  2. Implemented new practices;
  3. Implemented new guidelines/policies;
  4. Participated in joint projects with other institution;
  5. Obtained new membership in national/international organizations;
  6. Increased international visibility;
  7. Team work and sharing knowledge with new colleagues and peers;
  8. Gained new perspectives on TB lab organization and activity (staff, patient management, reporting, quality control etc.);
  9. other (please specify)_____________________________________________~~­­­­­­­­­­~~

1. Has your laboratory ever been visited by a team of Support Experts
2. Yes
3. No

IF YOU HAVE ANSWERED YES PLEASE GO TO QUESTION 10

IF YOU HAVE ANSWERED NO PLEASE GO TO QUESTION 12

1. What did gain your laboratory from the visit
   1. Written Report
   2. Verbal Report
   3. List of Recommendations
   4. Support provided on implementation of recommendations
   5. None
   6. Other (specify) ­­­­­­­­­_____________________________________
2. Did your laboratory take any action based on the findings/recommendations
   1. Yes (please specify)
   2. No
   3. I don’t know

1. Please rate the benefits of the Support Expert training program for the ERLTB-Net (0 no benefits; 10 – significant benefits)

____________________________________­­­­­­­­­­­­­­

1. Please rate the benefits of the Support Expert program for the TB diagnostic community within the EU (0 – no benefits; 10 – significant benefits)

­­­­­­____________________________________

1. In your opinion, how could the existing training programme be improved

_____________________________________________________________

_____________________________________________________________­­­­­­­­­­
