# Supplementary material for: European Union training programme for tuberculosis laboratory experts: design, contribution and future direction
Source: BMC Health Serv Res. 2020 May 11;20:413. doi: 10.1186/s12913-020-05240-3 (PMC7212721; doi:10.1186/s12913-020-05240-3)
Supplement: Supplementary file 4 — Additional file 4. Additional questionnaire on Missions and benefits for the laboratories for ERLTB-Network members and trained Support Experts. [file 12913_2020_5240_MOESM4_ESM.docx]

**Additional File 4:**

**Additional questionnaire on missions and benefits for the laboratories for ERLTB-Net members and trained Support Experts**

1. Please provide details of changes initiated and/or new technologies/methods implemented by yourself and/or your colleagues in your laboratory during or upon completion of your training as a Support Expert.

**Give examples and use as much space as needed**.

1. Please provide details on the type of mission you participated in eg. Country/laboratory assessment, exchange visit and who commissioned the mission eg. requested by ECDC, WHO/other organizations.

**Give examples and use as much space as needed**.
