# Supplementary material for: European Union training programme for tuberculosis laboratory experts: design, contribution and future direction
Source: BMC Health Serv Res. 2020 May 11;20:413. doi: 10.1186/s12913-020-05240-3 (PMC7212721; doi:10.1186/s12913-020-05240-3)
Supplement: Supplementary file 5 — Additional file 5. Additional topics suggested by participants for inclusion in the training programme. [file 12913_2020_5240_MOESM5_ESM.docx]

**Additional File 5:**

**Details on the additional topics suggested by participants for inclusion in the training programme**

- MIRU-VNTR, Whole Genome Sequencing and Bioinformatics
- MALDI-TOF Mass Spectrometry
- Refugees screening for latent TB and prophylactic treatment
- Financial sources to run the lab and how to best prioritize lab diagnostics from a TB-control perspective
- Research study design and brainstorming
- Building successful collaborations
- Components of a high-quality network of TB laboratories
- Capacity building for operational research
- Non-tuberculous mycobacteria and DST
