# Supplementary material for: European Union training programme for tuberculosis laboratory experts: design, contribution and future direction
Source: BMC Health Serv Res. 2020 May 11;20:413. doi: 10.1186/s12913-020-05240-3 (PMC7212721; doi:10.1186/s12913-020-05240-3)
Supplement: Supplementary file 6 — Additional file 6. Proposals from ERLTB-Net members to enhance the training programme. [file 12913_2020_5240_MOESM6_ESM.docx]

**Additional File 6:**

**Proposals from ERLTB-Net members to enhance the training programme**

- A staff exchange or longer visit in another lab
- Online training sessions for different topics
- It is important to learn how the data collected from different expensive typing methods implement in valuable way, how to use this knowledge beneficially in everyday work.
- More practical work for methods that are not used in all laboratories.
- Involve medical doctors and less young people since in my country young people do not have permanent position and thus they will be trained but then move away
- More focus on the twinning programmes between countries
- Relationships already established between labs should be further supported
- The training programme should be continued so that young colleagues have the opportunity to gain experience
- Persons that visit to give their expertise should come from laboratories with high standards regarding the variety of methods, quality, accreditation and experience
- Keep involving the experts in future meeting
- To have time for more practical work and discussions
